# Physical activity in adulthood: genes and mortality

**DOI:** 10.1038/srep18259

**Published:** 2015-12-15

**Authors:** Sira Karvinen, Katja Waller, Mika Silvennoinen, Lauren G. Koch, Steven L. Britton, Jaakko Kaprio, Heikki Kainulainen, Urho M. Kujala

**Affiliations:** 1 Department of Biology of Physical Activity, University of Jyväskylä, P.O. Box 35, FI-40014 University of Jyväskylä, Jyväskylä, Finland; 2 Department of Health Sciences, University of Jyväskylä, P.O. Box 35, FI-40014 University of Jyväskylä, Jyväskylä, Finland; 3 Department of Anesthesiology, University of Michigan Medical School, 2021 BSRB, 109 Zina Pitcher Place, Ann Arbor, Michigan, 48109-2200 USA; 4 Department of Molecular & Integrative Physiology, University of Michigan Medical School, 2021 BSRB, 109 Zina Pitcher Place, Ann Arbor, Michigan, 48109-2200 USA; 5 Department of Public Health, P.O. Box 41, FI-00014 University of Helsinki, Helsinki, Finland; 6 Institute for Molecular Medicine (FIMM), P.O. Box 20, FI-00014 University of Helsinki, Helsinki, Finland; 7 Department of Health, National Institute for Health and Welfare, P.O. Box 20, FI-00271 Helsinki, Finland

## Abstract

Observational studies report a strong inverse relationship between leisure-time physical activity and all-cause mortality. Despite suggestive evidence from population-based associations, scientists have not been able to show a beneficial effect of physical activity on the risk of death in controlled intervention studies among individuals who have been healthy at baseline. On the other hand, high cardiorespiratory fitness is known to be a strong predictor of reduced mortality, even more robust than physical activity level itself. Here, in both animals and/or human twins, we show that the same genetic factors influence physical activity levels, cardiorespiratory fitness, and risk of death. Previous observational follow-up studies in humans suggest that increasing fitness through physical activity levels could prolong life; however, our controlled interventional study with laboratory rats bred for low and high intrinsic fitness contrast with these findings. Also, we find no evidence for the suggested association using pairwise analysis among monozygotic twin pairs who are discordant in their physical activity levels. Based on both our animal and human findings, we propose that genetic pleiotropy might partly explain the frequently observed associations between high baseline physical activity and later reduced mortality in humans.

Intervention studies in humans show several positive effects of exercise on physical performance and health-related metabolism^1^. Also, in humans, observational follow-up studies report an association between high baseline levels of leisure-time physical activity and low all-cause mortality^2^. However, a causal relationship between adulthood physical activity and mortality/lifespan has not been confirmed, either in randomized controlled intervention studies, with initially healthy individuals, or in animal experiments^3^,^4^,^5^,^6^. High cardiorespiratory fitness is shown to be a strong predictor of reduced mortality, both in humans^7^,^8^ and in rats^9^. Keeping in mind that fitness measurements may have higher accuracy than measurements of physical activity levels, cardiorespiratory fitness (maximal oxygen consumption, VO_2_max) predicts mortality to a greater extent than physical activity level (metabolic equivalents, MET), when both are analysed in the same study^10^. Additionally, there is no consistent causal relation between (non-voluntary) occupation-related physical activity and subsequent mortality^11^,^12^. Thus, we lack definitive evidence on the acquired effect of physical activity on longevity. Based on past studies on elite athletes and twins, we previously suggested that genetic pleiotropy may explain at least some of the observed association between high physical activity and later morbidity or mortality^13^,^14^. Here, to increase our understanding of associations between genes, physical activity, and mortality, we carried out two studies. First, we conducted a controlled intervention study on the effects of voluntary running during adulthood on lifespan in two rat strains selectively bred for low and high intrinsic capacities for running (LCR and HCR, respectively). These two widely-segregated genetically heterogeneous rat strains have a 28–45% divide in median lifespan strongly predicted by a differential in VO_2_max across lifespan and are thus, well-suited for testing the longevity effects accrued from voluntary physical activity^15^. We chose to study female rats, because in contrast to males, female rats are known to compensate for increased physical activity with an increase in food intake^16^,^17^. Thus, female rats provided the opportunity to test the independent effects of exercise on mortality, without interference from calorie restriction, which is known to increase lifespan^18^.

Second, we conducted a long-term observational study in humans, with pairs of same-sex dizygotic (DZ) and monozygotic (MZ) twins. This study included a 15-y segment, where we recorded participation in physical activity, and a 23-year follow-up to evaluate mortality. Because MZ twins are genetically identical at the DNA sequence level, genetic factors were controlled in our activity-discordant, co-twin control analysis of MZ pairs. DZ twins share, on average, half of their segregating genes. Thus, genetic factors may influence physical activity discordances in DZ twin pairs. However, in most cases, both individuals in a pair of twins (MZ and DZ) share the same childhood environment; therefore, the childhood home environment is considered a controlled factor.

## Results

### Animal study

**Figure 1:**
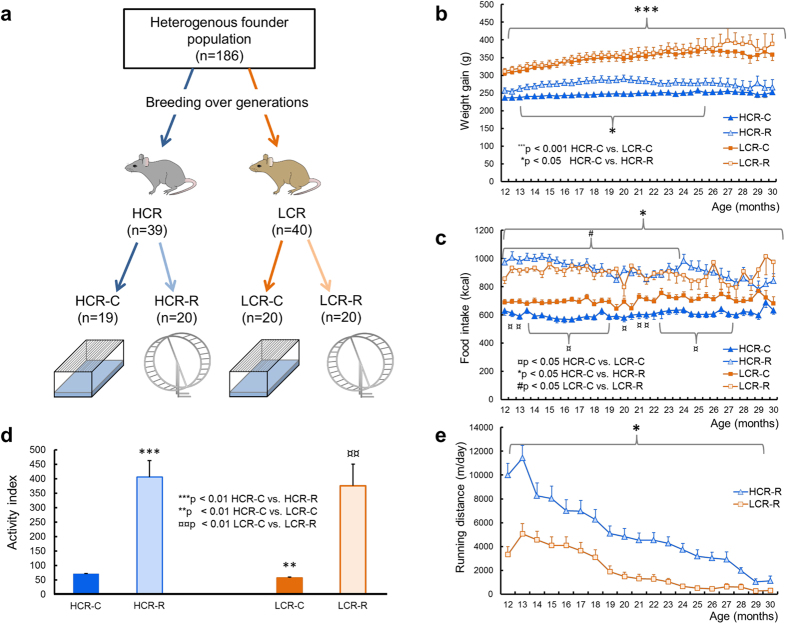
Rat study protocol and measurements. (**a**) Schematic of study protocol. Rats were bred for high (HCR, blue) or low (LCR, orange) intrinsic fitness, then assigned to control (C) or running (R) subgroups. (**b**) Body weights from ages 12 to 30 months. Rats per group: 12 months: HCR-C = 18, HCR-R = 15, LCR-C = 20, and LCR-R = 20; 21 months: HCR-C = 19, HCR-R = 17, LCR-C = 18, and LCR-R = 13; 30 months: HCR-C = 12, HCR-R = 8, LCR-C = 7, and LCR-R = 3. **(c)** Food intakes from ages 12 to 30 months. Rats per group: same as in **b)**, except at 12 months: HCR-C = 16. (**d**) Average daily spontaneous activity measured over 3 days between 13 and 15 months of age (activity index). Rats per group: 13 months: HCR-C = 18, HCR-R = 12, LCR-C = 20, and LCR-R = 11; 15 months: HCR-C = 19, HCR-R = 13, LCR-C = 19, and LCR-R = 10. **(e**) Average running distance per day. Rats per group: 12 months: HCR-R = 15 and LCR-R = 15; 21 months: HCR-R = 16 and LCR-R = 14; 30 months: HCR-R = 4 and LCR-R = 2. Error bars represent SEMs. Figure was drawn by Sira Karvinen.

The HCR/LCR contrasting rat model system for intrinsic fitness capacity is the result of an over 15 year two-way artificial selection experiment described previously^19^. For the current study, we obtained 79 female rats (39 HCR and 40 LCR) and began testing when the rats were ~9 months old. Figure 1a shows the study design. We randomly assigned LCR and HCR rats to control (C) and running (R) subgroups matched within strain for body weight and fitness capacity: 19 HCR-C and 20 LCR-C, maintained in standard cages; and voluntary runners: 20 HCR-R and 20 LCR-R, maintained in cages equipped with a running wheel to permit voluntary running throughout adulthood. Figure 1b,c show details on measured body weight and food intake for all four study groups across 12–30 months of age. As previously shown, LCR rats weigh significantly more than HCR rats (age 3 months, P < 0.001, data not shown), and this difference remained significant throughout adult life (Fig. 1b, HCR-C vs. LCR-C, 12–30 months, P < 0.001). Food intake was significantly greater in runner groups compared to control for both LCR and HCR (Fig. 1c, HCR-C vs. HCR-R, 12–30 months, P < 0.05).

We measured the natural total spontaneous physical activity (horizontal and vertical movements) every three months throughout each rat’s lifespan using a custom designed force-plate system^20^. The activity index was clearly higher among the runners than among the corresponding controls (Fig. 1d). Consistent with previous reports, the HCR-C group exhibited higher spontaneous activity than the LCR-C group by 23% (Fig. 1d, *P* < 0.01). In addition, for cages equipped with running wheels, the average wheel distance per day was longer in HCR-R than in the LCR-R group at time points from 12 to 29 months (Fig. 1e, *P* < 0.05).

Because physical activity is known to have positive effects on glucose metabolism, we measured insulin sensitivity at baseline (age 9 months) and after 1 year of intervention (age 21 months). At baseline, all four rat groups had similar insulin sensitivity, based on the HOMA-IR index^21^. After one year of voluntary running, the LCR-R group had a significantly lower HOMA-IR index than the LCR-C group (3.5 ± 3.0 vs. 5.7 ± 2.8, *P* < 0.05). In HCR groups, there was no difference in the HOMA index between groups (4.3 ± 3.1 vs. 4.6±3.0, respectively), which is consistent with the fact that HCRs represent a healthier phenotype compared to LCRs, having lower risk of metabolic diseases even without physical training.

**Figure 2:**
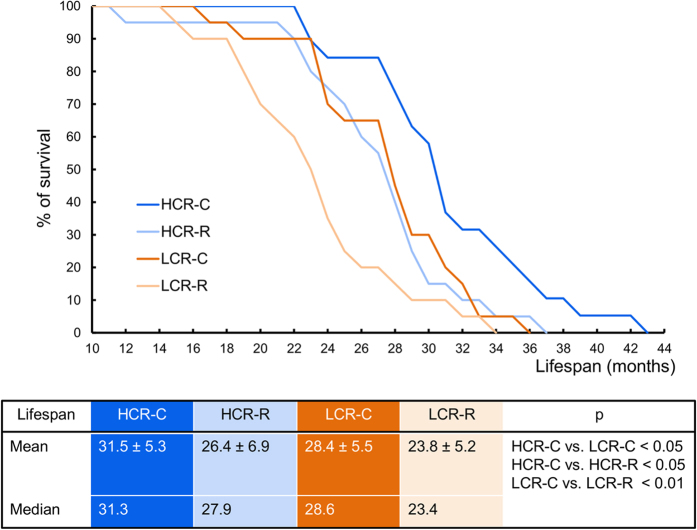
Effects of genetic background and environment on lifespan. Control rats (C) had longer lifespans than rats in the runner groups (R) of the same strain (HCR-C vs. HCR-R, *P* < 0.05 and LCR-C vs. LCR-R, *P* < 0.01). Mean lifespans were also significantly different between rat strains (HCR-C vs. LCR-C, *P* < 0.05). Values in the table show means±SDs.

As expected, the Cox proportional hazards model showed that LCR rats had a higher risk of death than HCR rats, the hazard ratio (HR) for LCR compared to HCR rats adjusted for running group and age at randomization being 1.7 (95% CI: 1.1–2.6, *P *= 0.028). Interestingly, when combined, the pool of LCR and HCR runner rats had an increased risk of death compared to control groups, with a HR of 2.1 (95% CI: 1.3–3.4; *P* < 0.001). This finding persisted after adjustments for strain, age at randomization, and body weight at 9 months of age (multivariate-adjusted HR = 2.3, 95% CI: 1.4–3.6; *P* < 0.001). The decreased survivability for runners vs. controls was similar for both HCR and LCR strains. The survival curves in Fig. 2 shows the mean lifespan among runners was consistently 16% shorter than among controls in both the HCR (mean 26.4 vs. 31.5 months, *P* < 0.05) and LCR (23.8 vs. 28.4 months, *P *< 0.01) groups. The deaths caused by development of spontaneous tumours with aging did not explain the group differences in lifespan (21–45% of deaths within group).

### Human study

**Figure 3:**
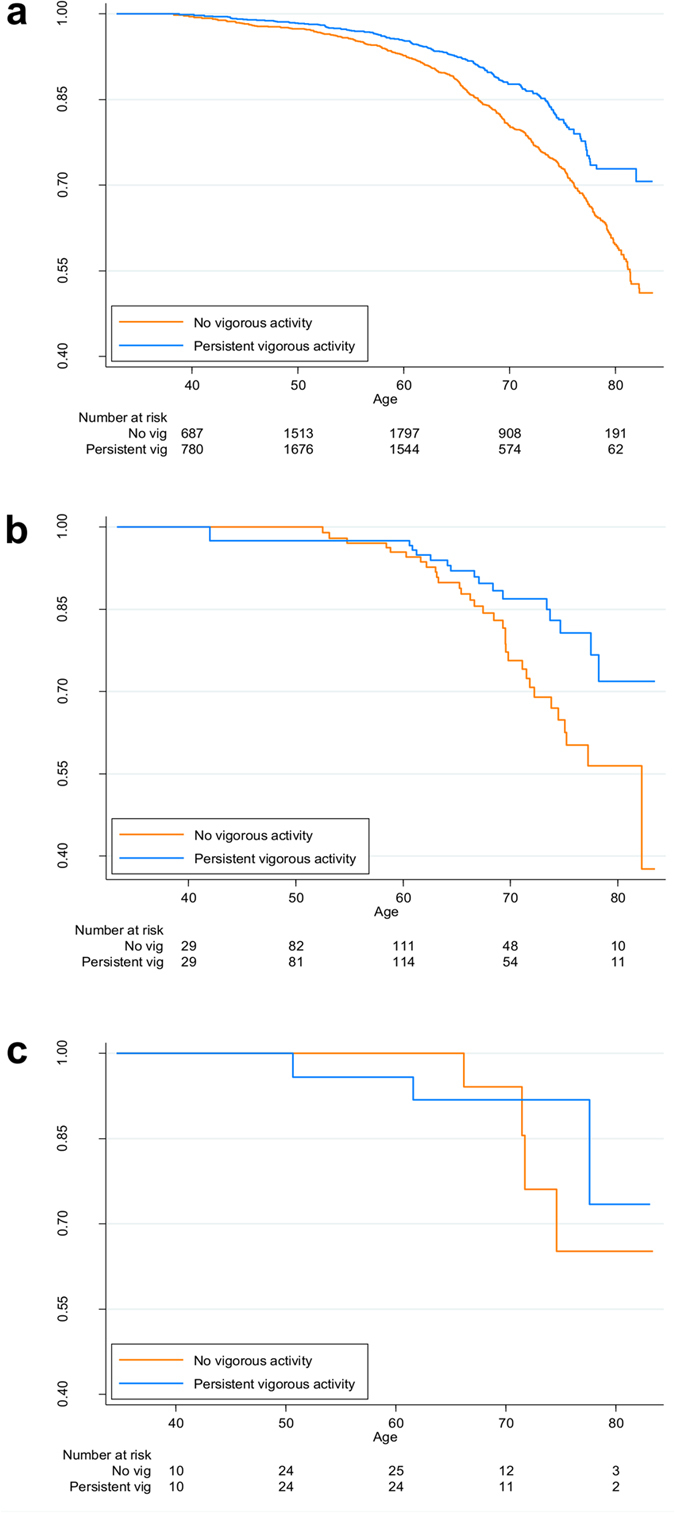
Kaplan-Meier survival curves of mortality in the human study. Follow-up started from the date of the 1990 questionnaire response to the end of July 2013. Groups comprised individuals with no vigorous activity (orange) vs. those with persistent vigorous activity (blue) at baseline (start of follow-up). (**a**) Survival of 2428 individuals with no vigorous activity and 2145 individuals with persistent vigorous activity; (**b**) survival of 134 discordant DZ twin pairs; (**c**) survival of 34 discordant MZ twin pairs.

The prospective Finnish Twin Cohort^22^ includes all same-sex twin pairs born in Finland before 1958. Physical activity was measured with a structured questionnaire. We used persistence and changes in vigorous physical activity during the years 1975, 1981, and 1990 as baseline predictors of mortality. Altogether, 11 325 twin individuals (4190 complete twin pairs) answered the required physical activity questions for all three baseline time points (for more details of the cohort, see Table S1). A Cox proportional hazards model was used to analyse mortality, starting from the 1990 response date and ending July 31, 2013. Altogether, 458 deaths were observed among individuals that had performed no vigorous activity at baseline (in 1975, 1981, and 1990) and 201 deaths among individuals that persistently performed vigorous activity. An individual-based analysis showed that, compared to individuals with no vigorous activity, individuals with persistent vigorous physical activity showed decreased mortality (in 1975, 1981, and 1990); the age- and sex-adjusted HR of death was 0.55 (95% CI: 0.46–0.64) and the HR adjusted for sex, age, education level, smoking status in 1990, alcohol use (grams per day) in 1990, BMI in 1990, work activity, and health status in 1990 was 0.73 (0.61–0.88) (Fig. 3a and Table S2).

Of the 4190 same-sex twin pairs (MZ = 1388, DZ = 2547, 255 unknown zygosity), we identified 179 (4.3%) persistently discordant for participation in vigorous physical activity. These activity-discordant twin pairs comprised 2.4% (34 of 1388) of all MZ pairs and 5.3% (134 of 2547) of all DZ pairs (*P* < 0.001; Fisher’s exact test for a difference in persistent discordances between MZ and DZ pairs). A pairwise analysis of these 179 twin pairs showed that the mortality HR for persistent vs. non-persistent vigorous activity was 0.65 (0.46–0.91), and after adjusting for all covariates including health status, the HR was 0.72 (0.48–1.07). Consistent with our previous twin analysis, which was based on a shorter period of physical activity discordances^23^, the activity-discordant DZ pairs showed a difference in mortality (Fig. 3b, HR = 0.58, 95% CI: 0.39–0.88). However, no difference was observed in the pairwise analysis of the smaller group of activity-discordant MZ pairs (Fig. 3c, HR = 1.00, 95% CI: 0.52–1.94). The heritability of physical activity (see below) contributed to the statistical power of the analysis among MZ pairs.

To describe the total volume of physical activity performed during leisure time, we calculated a metabolic equivalent (MET) index expressed as the sum of leisure MET-hours per day at each time-point (1975, 1981, and 1990). The means of the three MET index values showed that MZ pairs (0.54) had a higher Intraclass Correlation Coefficients (ICC) than DZ pairs (0.26). This result indicates that the differences in the genetic component of the total variance influenced the long-term levels of physical activity compared to the environmental component. Using standard techniques for genetic modelling^24^ (AE model), we estimate the narrow-sense heritability for physical activity to be 53% (95% CI: 46–59%).

Due to the low number of deaths among MZ twin pairs we repeated the pairwise analyses among DZ and MZ pairs separately using cohort members who were discordant for vigorous physical activity in 1975 and 1981 (similar criteria as in our primary analysis but shorter PA discordance at baseline). This material also included older cohort members than in our primary analysis. This dataset included 778 DZ and 231 MZ vigorous activity discordant twin pairs. Among these DZ pairs there were 204 deaths and among MZ pairs 55 deaths during follow-up (between 1981 and 31^st^ August 2013). Unadjusted pairwise HR for death among active compared to inactive members for DZ pairs was lower (HR 0.58 [95% CI 0.46–0.74]) than among MZ pairs (0.85 [0.56–1.30]. When this secondary analysis was repeated in the baseline-healthy subgroup the HRs were 0.64 (0.45–0.89) for DZ pairs and 1.05 (0.58–1.88) for MZ pairs, respectively.

We also performed an individual-based analysis of how work-related (non-voluntary) physical activity affected mortality. We found that individuals with persistently non-sedentary work had a higher risk of death than those with persistently sedentary work (age- and sex-adjusted HR = 1.15, 95% CI: 1.00–1.32; *P* = 0.046). Additional analyses that adjusted for other covariates (e.g. sex, age, education, smoking status, alcohol consumption and BMI) attenuated the HR. Also, multivariate models that analysed the persistence or change in work-related physical activity during 1975–1990 did not show any statistically significant associations with subsequent mortality. Moreover, no differences for the risk of death by work-related physical activity were observed in the pairwise analyses of all twin pairs.

## Discussion

The main findings of this study along with their interpretations are summarized in Table S3. Taken together, our results are consistent with previous data on rodents^15^,^25^,^26^ and humans^27^,^28^, which indicated that genetic predisposition plays a significant role in exercise participation. These results are also consistent with our previous suggestion^14^ that genetic pleiotropy may partly explain the associations observed between high physical activity and mortality in our past epidemiological studies, which called for high quality intervention studies to analyse the true effects of physical activity on morbidity and mortality among initially healthy individuals. Our results also support the notion that inherited aerobic capacity is a predictor of longevity^9^,^13^, but further study in both animals and humans is required to determine whether this is true for the portion of aerobic capacity enhanced by vigorous physical activity. Our findings are also consistent with previous studies that show positive effects of physical activity on glucose metabolism in rodents and human twins^29^. However, vigorous physical activity does not improve longevity in twins^23^ or rodents, particularly when commenced in maturity^3^,^31^. It is to note that randomized controlled trials show that vigorous physical activity has other health benefits such as improvement of both self-reported and objectively reported physical functioning and reduction of depression^1^,^32^,^33^.

With this work as a foundation, we anticipate a variety of future investigations. Examples include large scale, randomized controlled intervention trials on the effects of physical activity, collaborative twin studies with larger sample sizes, Mendelian randomization analyses, and studies on genetic pleiotropy, after obtaining more knowledge on susceptibility genes. Also, there are limitations in using questionnaires as a measure of physical activity level, and future studies should combine self-reported PA data with proper objective monitoring of physical activity including specific causes of death as outcomes. In our animal experiment, divergence in physical activity started in early adulthood, which is typically the case in physical activity-discordant MZ twin pairs^30^. Our finding covers vigorous physical activity started at adulthood, but low intensity leisure-time physical activity such as walking and vigorous physical activity started during childhood may have different effects. Thus, it will be critical to determine whether physical activity affects lifespan differently when commenced early in life compared to starting later in adult life^31^,^34^,^35^.

## Methods

### Animal study

#### Animal strains

The HCR/LCR contrasting rat model system was produced with two-way artificial selection, starting from a founder population of 186 genetically heterogeneous rats (N:NIH stock), as described previously^19^ (Fig. 1a). Briefly, endurance running capacity was assessed on a treadmill and the total distance run during the test was used as a measure for intrinsic fitness capacity. Rats with the highest running capacity from each generation were bred to produce the HCR strain, and rats with the lowest capacity were bred to produce the LCR strain. For the study protocol described here, 79 female rats (39 HCR and 40 LCR) were obtained from generations 23–27. Each rat was phenotyped for fitness capacity at the University of Michigan (Ann Arbor, Michigan, USA) with a speed-ramped treadmill running test (15° slope, initial velocity of 10 m × min^−1^, increased 1 m × min^−1^ every 2 min), when rats were ~3 months of age. Clear differences were observed in running test results (HCR 2164 ± 394 m vs. LCR 302 ± 49 m, *P* < 0.001).

After the rats arrived and habituated in Finland, we collected fasting blood samples from untrained animals (mean age 9 months), and again one year after the start of the voluntary running intervention (age 21 months). Then, each rat was carefully followed to measure lifespan. All rats were maintained in an environmentally controlled facility (12/12 h light-dark cycle, 22 °C) with water and standard food *ad libitum*.

#### Intervention

Before testing, up to the age of 9 months, rats were housed 2/cage in standard cages. After collecting the first blood samples (age 302 ± 38 days), each group (HCR and LCR) was divided into two subgroups matched for body weight and maximal running capacity; two subgroups were placed in standard cages (controls: HCR-C and LCR-C) and two subgroups were placed in cages with running wheels (voluntary running groups: HCR-R and LCR-R; Fig. 1a). During the voluntary running intervention, rats in all four groups were housed 1/cage. Voluntary running was followed through a computerized system throughout lifespan. Follow-up started on the day of randomization and ended at death.

Animals were euthanized when any one of the predetermined humane end point criteria were observed. These criteria were: movement disabilities, difficulty maintaining an upright position, assuming a crouched position for >48 h, labored breathing, dehydration, severe loss of body mass (>20% total body mass), chronic diarrhea or constipation for >48 h, test results that indicate loss of internal organ function, any prolonged leak from a body orifice, self-harming behaviour, and unresponsiveness to external stimuli.

#### Measurements of spontaneous activity

Total spontaneous physical activity was measured every three months for four days throughout each rat’s lifespan. For this purpose, we used ground reaction force recordings, as described previously^20^. From that data, we calculated the activity index^20^,^36^ as an average sum of three full days from 8 a.m. to 8 a.m. Data shown is an average value of measurements from time points 13 and 15 months of age.

#### Measurement of body weight and energy intake

The body weights and energy intakes of rats were followed throughout the study by weighing the rats and the amount of food consumed every second week. The energy intake was calculated based on the estimated energy content of the food, according to the manufacturer (R36, Lactamin AB).

#### Fasting blood samples

Fasting blood samples were collected at day time, after 5 h fasting, at ages 9 and 21 months. The group sizes were reduced between 9 and 21 months as follows: HCR-C: 19 to 17; HCR-R: 20 to 17; LCR-C: 20 to 18; and LCR-R: 20 to 15. Before collecting blood samples, all running wheels were blocked for 5 h to disable wheel movement. This action precluded any potential acute effects of running on the measured parameters. Blood glucose was measured in fresh samples (HemoCue Glucose 201 RT). Insulin was measured with ELISA from frozen (-80 °C) serum samples (Mercodia, Rat Insulin ELISA). HOMA-IR was calculated as the product of fasting glucose and insulin levels, divided by a constant^21^, with the following equation:

(Glucose (mmol/l) * Insulin (μg/l))/2.430.

#### Ethics statement

Experimental procedures with rats conformed to the European Guidelines for the Care and Use of Laboratory Animals (directive 2010/63/EU) and were approved by the National Animal Experiment Board, Finland (Permit number ESAVI-2010-07989/Ym-23).

#### Statistical Analyses

We performed statistical analyses using IBM SPSS for Windows 22.0 (SPSS Inc., Chicago, IL, USA). The Shapiro-Wilk test was used to investigate within-group normality for a given parameter of interest. Levene’s test was used to assess the homogeneity of the variance assumption. When the assumptions were met, statistical comparisons of parameters between rat groups were performed with the T-test. *P*-values less than 0.05 were considered statistically significant.

Mortality analyses were conducted in individual study groups (e.g., HCR-R vs. LCR-R); in pooled groups (HCR vs. LCR); and according to training (e.g., HCR-R vs. HCR-C). Hazard ratios (HR) and 95% confidence intervals (CI) were calculated with the Cox proportional hazard model with the StataIC13 statistical package. Follow-up started on the day of randomization and ended at death. Results were adjusted for age at randomization and body weight.

## Human study

### Twin cohort

The Finnish Twin Cohort retains records of all same-sex twin pairs born in Finland before 1958, if both co-twins were alive in 1967^22^. In 1975, a baseline questionnaire was sent to twin pairs that were both alive at that time. A second questionnaire was sent in 1981 to all twin pairs; and a third questionnaire was sent in 1990 to all twins aged 33–60 years that had responded to at least to one of the earlier questionnaires. Among those with known addresses (93.5% of subjects) in 1975, the response rate for twin pairs was 87.6%. The response rate among respondents in 1975 that were alive in 1981 was 90.7%. The individual response rate was 77.3% in 1990^37^. The determination of zygosity was based on an accurate, validated questionnaire^38^.

For the present study, inclusion criteria were: complete data on leisure-time physical activity required for calculating the metabolic equivalent (MET) index, from three postal surveys conducted in 1975, 1981, and 1990. A total of 11 325 individuals (5113 males and 6212 females) met these criteria for all three time points, including 4190 complete twin pairs (1388 MZ pairs, 2547 DZ pairs, and 255 pairs with unknown zygosity). The physical activity levels and mortality of twins did not differ between pairs with one respondent (ineligible for pairwise analyses) and pairs with two respondents (eligible for pairwise analyses).

### Assessment of predictors and potentially confounding variables

The questionnaires mailed in 1975, 1981, and 1990 included questions on physical activity performed during leisure time, work-related physical activity, weight, height, occupation, alcohol use, smoking, and physician-diagnosed diseases. For the current study, participation in vigorous leisure time physical activity (voluntary) and work-related physical activity (less voluntary) in 1975, 1981, and 1990 were used as separate baseline predictors of mortality.

### Leisure-time physical activity

Leisure physical activity habits were assessed with identical validated questions on surveys conducted in 1975 and 1981^40^,^40^ and with modified questions on the survey conducted in 1990^41^. Assessments of vigorous physical activity were based on a single set of intensity categories applied at all three time points; these were: 1) walking, 2) alternately walking and jogging, 3) jogging (light running), and 4) running. Vigorous activity was defined as levels 2, 3, or 4 (all more intensive than normal walking), performed at least 3–5 times/month in 1975 and 1981, or at least for 30 min each week in 1990. Then, a five-category vigorous activity variable was created to assess changes/persistence over time. Category 1 was ‘persistent vigorous activity’, defined as vigorously active at all three time points (1975, 1981, and 1990); conversely, category 5 was ‘no vigorous activity’ defined as no vigorous activity at all three time points. Intermediate categories were mixed participation in vigorous activity over time (1975, 1981, 1990, respectively), as follows: ‘increased’ (no, no/yes, yes), ‘decreased’ (yes, yes/no, no), and ‘changed’ ([yes, no, yes] or [no, yes, no]). This five-category vigorous activity variable was used as the main mortality predictor for the human study.

To characterize the amount of long-term leisure time physical activity performed, we calculated the MET index, based on a series of structured questions that covered leisure physical activity and any physical activity performed during the journey to and from work^39^. We calculated the activity MET index by assigning a multiple of resting metabolic rate to each activity and by calculating intensity x duration x frequency of activity. The MET index was expressed as the sum of leisure MET-hours per day at each time-point (1975, 1981, and 1990). Finally, we calculated a mean MET index for long-term leisure-time physical activity; i.e., the MET indexes for 1975, 1981, and 1990 were added together and divided by 3.

### Work-related physical activity

Work-related physical activity was a categorical variable evaluated with a four-point ordinal scale^23^. The question was as follows: “What kind of work did/do you do in your present job or the job you last had?” The answers were: 1) mainly sedentary work, which requires very little physical activity; 2) work that involves standing and walking, but no other physical activity; 3) work that requires standing and walking, and in addition, requires lifting and carrying; or 4) heavy physical work. Answer 1 was classified as sedentary work, and answers 2, 3, or 4 were classified as non-sedentary work. Then, a five-category work-related activity variable was created that covered the three measurement points, as described for participation in vigorous leisure-time physical activity (see above). Those categories were: persistent sedentary work, persistent non-sedentary work, increased, decreased, or changed work-related physical activity.

### Confounding variables

For the present study, body mass index (BMI), smoking status, use of alcohol, health status (all reported in 1990), and self-reported education level (reported in 1981) were tested as covariates. The level of education was assessed in the 1981 questionnaire by asking what kind of schooling the respondent had completed^37^. The answers were converted into a nine-class variable of years of schooling for the analyses. BMI (kg/m^2^) was calculated from self-reported height and weight. Smoking status was determined from responses to detailed questions on smoking history, coded into four categories (never smoked, former smoker, occasional smoker, and current (daily) smoker)^42^. Alcohol use was expressed in units of alcohol grams consumed per day^43^. According to the 1990 questionnaire, individuals were considered to have a somatic illness, when he ⁄ she had (i) any self-reported disease diagnosed by a physician, or (ii) a self-reported life event related to a serious injury or illness, or (iii) a self-reported permanent work disability^37^. Other subjects were classified as healthy. The cohort included a total of 4846 twins who were healthy in 1990; 91.4% of them had lived together with their co-twin until the age of 16 years.

### Ethics statement

An earlier statement from the ethics committee of the Department of Public Health, University of Helsinki covered our current study. The study was conducted according to the declaration of Helsinki. All the participants gave their informed consent by providing questionnaire responses. The participants were provided regular feedback on the purpose and conduct of the study and were informed that they may withdraw from the study at any time.

### Statistical analysis

Mplus version 7 was used to estimate Intraclass Correlation Coefficients (ICC) and to partition the total variance into genetic and environmental components^44^ with standard techniques^24^. All-cause mortality during follow-up was analysed. The exact dates of death, causes of death, and emigration from Finland were available from the Population Register Centre of Finland. A total of 243 177 person-years were accumulated during follow-up, from the 1990 individual response date to the end of July 2013, the date of emigration, or death. During that period, 1478 individuals died at a mean age of 63.6 years (range 37.1 to 82.8 years). First, we analysed individual-based mortality in relation to leisure-time physical activity participation, adjusted for age and sex. We calculated HRs and 95% CIs with the Cox proportional hazard model, and we applied clustering for family members. Next, we adjusted the model for baseline levels of education, smoking status, alcohol use, BMI, and work-related activity, by adding one covariate at a time to the model. These adjustments did not significantly change the HRs; therefore, three different models are shown in the Table S2. Additionally, a fourth model is shown, where health status was added to the full model. Individuals that did not participate in vigorous activity were used as the reference group in all analyses of leisure time physical activity. In addition, pairwise analyses were performed with the same models for all twin pairs. We analysed all twins at once, and then, MZ and DZ pairs separately. We used the identical analysis strategy for assessing work-related physical activity categories, except we did not adjust for work-related physical activity. Data were analysed with the StataIC13 statistical package (Stata Corp, College Station, Texas, U.S.A.).

## Additional Information

**How to cite this article**: Karvinen, S. *et al.* Physical activity in adulthood: genes and mortality. *Sci. Rep.* **5**, 18259; doi: 10.1038/srep18259 (2015).

## Supplementary Material

Supplementary Information
