# Supplementary material for: Physical activity in adulthood: genes and mortality
Source: Sci Rep. 2015 Dec 15;5:18259. doi: 10.1038/srep18259 (PMC4678877; doi:10.1038/srep18259)
Supplement: Supplementary Information [file srep18259-s1.pdf]

Supplementary Information Karvinen et al.

Physical activity in adulthood: genes and mortality

Sira Karvinen[*], Katja Waller, Mika Silvennoinen, Lauren G. Koch, Steven L. Britton, Jaakko Kaprio, Heikki Kainulainen & Urho M. Kujala

Table S1. Baseline characteristics according to whether vigorous physical activity was absent, persistent, or changed between three baseline time points (1975, 1981, and 1990)

Variable	No vigorous activity	Persistent vigorous activity	Increased vigorous activity	Decreased vigorous activity	Changed vigorous activity	*P* value*
All n (%)	2 428 (21.4)	2 145 (18.9)	4 285 (37.8)	927 (8.2)	1 540 (13.6)	
Male n (%)	1 093 (21.4)	1 212 (23.7)	1 626 (31.8)	533 (10.4)	649 (12.7)	
Female n (%)	1 335 (21.5)	933 (15.0)	2 659 (42.8)	394 (6.3)	891 (14.3)	
Monozygotic n (%)	707 (20.1)	682 (19.4)	1 349 (38.3)	316 (9.0)	470 (13.3)	
Dizygotic n (%)	1 563 (22.1)	1 321 (18.7)	2 684 (37.9)	552 (7.8)	959 (13.6)	
Age in 1990, years (mean ± SD)†	46.4 ± 8.3	43.6 ± 7.1	43.3 ± 7.4	45.7 ± 7.8	43.8 ± 7.6	< 0.001
BMI in 1990, kg/m^2 (mean ± SD)	25.6 ± 4.4	24.1 ± 3.1	24.0 ± 3.4	25.5 ± 3.9	24.5 ± 3.6	< 0.001
Alcohol consumption in 1990, grams/day (mean ± SD)	10.3 ± 18.1	10.3 ± 13.3	9.2 ± 14.5	10.9 ± 15.5	9.6 ± 14.4	0.09
Mean MET, 1975-1990, MET h/day (mean ± SD)	1.1 ± 0.8	5.2 ± 3.3	2.5 ± 1.6	2.6 ± 2.0	2.7 ± 1.6	< 0.001
Smoking status, current smoker, n (%)	727 (31.2)	381 (18.0)	1 056 (25.3)	248 (27.6)	365 (24.2)	< 0.001
Education 1981, at least the Finnish Matriculation Examination‡, n (%)	263 (10.8)	616 (28.7)	744 (17.4)	187 (20.2)	327 (21.3)	< 0.001
Work activity in 1990, sedentary work, n (%)	723 (30.7)	890 (41.8)	1 521 (36.1)	375 (41.0)	585 (38.5)	< 0.001

**P* values are from cluster-corrected regression analyses, adjusted for sex and age in 1990 for continuous variables, and from a symmetry test for categorical variables
†Age is calculated from the date that the questionnaire was returned in 1990 and the National population register information on date of birth
‡Equivalent to 12 years of education (primary and secondary education)

Table S2. Hazard ratios* for all-cause mortality from 1990 to July 31, 2013 for individual and pairwise twin analyses according to whether vigorous physical activity was persistent or changed between three baseline time points (1975, 1981, and 1990).

	**Individual analyses**				**Pairwise analyses among twins**			
	**age- and sex -adjusted**	**+ education† adjusted**	**Full model‡**	**Full model + healthy§**	**basic model**	**+ education† adjusted**	**Full model‡**	**Full model + healthy§**
**All***	n = 11 322	n = 11 313	n = 10 739	n = 10 480	n = 11 322	n = 11 313	n = 10 739	n = 10 480
Persistent activity	0.55 (0.46-0.64)	0.58 (0.49-0.69)	0.70 (0.58-0.84)	0.73 (0.61-0.88)	0.65 (0.46-0.91)	0.66 (0.47-0.93)	0.71 (0.48-1.05)	0.72 (0.48-1.07)
Increase	0.74 (0.65-0.84)	0.76 (0.67-0.87)	0.83 (0.72-0.96)	0.86 (0.75-0.99)	0.78 (0.60-1.02)	0.80 (0.61-1.04)	0.78 (0.58-1.05)	0.81 (0.59-1.10)
Decrease	0.88 (0.73-1.06)	0.91 (0.76-1.10)	0.93 (0.77-1.13)	0.95 (0.78-1.16)	0.96 (0.67-1.38)	0.97 (0.67-1.39)	0.87 (0.57-1.32)	0.86 (0.56-1.32)
Change	0.77 (0.65-0.91)	0.80 (0.67-0.95)	0.84 (0.70-1.01)	0.86 (0.72-1.03)	0.93 (0.67-1.29)	0.94 (0.67-1.31)	0.98 (0.67-1.42)	0.98 (0.67-1.45)
**MZ***					n = 3524	n = 3523	n = 3368	n = 3286
Persistent activity					1.00 (0.52-1.94)	0.90 (0.45-1.80)	0.89 (0.40-2.01)	1.01 (0.44-2.32)
Increase					1.04 (0.64-1.70)	1.06 (0.64-1.76)	1.14 (0.63-2.09)	1.30 (0.70-2.41)
Decrease					1.15 (0.58-2.31)	1.13 (0.55-2.31)	1.05 (0.43-2.56)	1.18 (0.48-2.89)
Change					1.27 (0.71-2.27)	1.23 (0.68-2.23)	1.33 (0.65-2.70)	1.44 (0.70-2.98)
**DZ***					n = 7076	n = 7068	n = 6688	n = 6530
Persistent activity					0.58 (0.39-0.88)	0.60 (0.39-0.92)	0.63 (0.39-1.02)	0.60 (0.36-0.99)
Increase					0.73 (0.53-1.01)	0.76 (0.54-1.06)	0.73 (0.50-1.06)	0.76 (0.51-1.12)
Decrease					1.03 (0.66-1.59)	1.06 (0.67-1.66)	0.89 (0.53-1.48)	0.84 (0.49-1.44)
Change					0.85 (0.56-1.30)	0.88 (0.57-1.35)	0.94 (0.58-1.52)	0.88 (0.53-1.46)

*Reference group in all analyses was a group that did not participate in vigorous activity during any of the three baseline time-points (1975, 1981, and 1990).

†Education level reported in 1981

‡Full model includes sex, age, education (9 categories), smoking status in 1990 (four categories), alcohol consumption (continuous, grams consumed in 1990 including abstainers), BMI in1990, and work activity

§Full model + Somatic health variable. The Somatic Disease Index (SDI) was assessed in the 1990 questionnaire, as follows: (i) any self-reported disease diagnosed by a physician, or (ii) a self-reported life event that caused serious injury or illness, or (iii) a self-reported permanent work disability

Table S3. Interpretation of the findings of different research questions.

Research question	Finding	Interpretation
Do rats with genetically determined high fitness have longer life expectancy than low fit rats?	Yes, rats with high fitness have significantly longer life expectancy than rats with low fitness.	In agreement with previous data on rodents and humans high aerobic fitness is a strong predictor of reduced mortality.
Does voluntary running started at adult age increase life expectancy in low fit or high fit rats?	No, voluntary running started at adult age significantly reduced life expectancy both among high fit and low fit rats.	In agreement with previous rodent studies exercise started at adult age did not reduce mortality or increase lifespan. However, there are studies suggesting that exercise started at young age may reduce mortality.
Do sequence level genetic differences influence the physical activity habits so that persistent discordances in participation in vigorous leisure-time physical activity are more common in dizygotic than monozygotic twin pairs?	Yes, the persistent discordances in participation in vigorous physical activity were significantly more common in dizygotic twin pairs (sharing 50% of their DNA sequence on average) than in monozygotic pairs (sharing 100% of their DNA sequence)	Genes have an influence on the persistent voluntary participation in vigorous leisure-time physical activity.
Does high work-related physical loading decrease mortality?	No, in our twins high work-related loading was not associated with reduced risk of death.	The lacking association between high work-related loading and later mortality is in agreement with previous epidemiological studies among individuals having been healthy at baseline.
Does high long-term participation in vigorous leisure-time physical activity predict mortality in twin individuals.	Yes, high long-term participation in vigorous leisure-time physical activity predicted significantly reduced mortality in twin individuals.	This finding is in agreement with epidemiological data, but causal relation between high physical activity and reduced mortality remains speculative as genetic pleiotropy may explain this association.
Does high long-term participation in vigorous leisure-time physical activity predict mortality in a pairwise analysis among activity discordant dizygotic twin pairs?	Yes, high long-term participation in vigorous leisure-time physical activity predicted significantly reduced mortality among active compared to inactive members of dizygotic twin pairs.	This finding is in agreement with epidemiological data, but causal relation between high physical activity and reduced mortality remains speculative as genetic pleiotropy may explain this association.
Does high long-term participation in vigorous leisure-time physical activity predict mortality in a pairwise analysis among activity discordant monozygotic twin pairs?	No, high long-term participation in vigorous leisure-time physical activity did not predict reduced mortality in pairwise analysis among monozygotic twin pairs. Possibly due to the sequence level genetic similarity it was even difficult to find pairs who were discordant for physical activity.	This finding is consistent with the lacking evidence from randomized controlled trials on baseline healthy individuals that high physical activity would reduce mortality. The finding is consistent with the hypothesis that genetic pleiotropy may explain at least some of the association between high physical activity and reduced mortality.
